# Turn Fast and Win: The Importance of Acyclic Phases in Top-Elite Female Swimmers

**DOI:** 10.3390/sports9090122

**Published:** 2021-08-31

**Authors:** Dennis-Peter Born, Joris Kuger, Marek Polach, Michael Romann

**Affiliations:** 1 Department for Elite Sport, Swiss Federal Institute of Sport Magglingen, 2532 Magglingen, Switzerland; joris.kuger@web.de (J.K.); michael.romann@baspo.admin.ch (M.R.); 2 Section for High-Performance Sports, Swiss Swimming Federation, 3063 Bern, Switzerland; 3 Department for Competitive Swimming, Czech Swimming Federation, 160 17 Prague, Czech Republic; marek.polach@czechswimming.cz; 4 Department of Social Sciences in Kinanthropology, Palacký University Olomouc, 771 47 Olomouc, Czech Republic

**Keywords:** analysis, biomechanics, coaching, data, training

## Abstract

The aim of the study was to investigate the effect of start and turn performances on race times in top-elite female swimmers and provide benchmarks for all performance levels, all swimming strokes, and all race distances of the European Short-Course Championships (EC). The individual races (*n* = 798) of all female competitors (age: 20.6 ± 3.9 years, FINA points: 792 ± 78) were video-monitored for subsequent analysis of start and turn performances. Benchmarks were established across all competitors of each event based on the 10th, 25th, 50th, 75th, and 90th percentiles. Start and turn performances contributed up to 27.43% and 56.37% to total race time, respectively. Mechanistic analysis revealed that the fastest swimmers had the lowest contribution of the acyclic phases to race time. Therefore, relative to their faster race times, these swimmers were even faster during starts and turns. Multiple linear regression analysis showed large effects of turn performance on 50, 100, 200, 400, and 800 m race times (β = 0.616, 0.813, 0.988, 1.004, and 1.011, respectively), while the effect of start performance continuously decreased the longer the race distance. As turn performance may be the distinguishing factor in modern short-course races, benchmarks should be used to set goals and establish training guidelines depending on the targeted race time.

## 1. Introduction

Recent race results show the diminishing performance gap between top-elite swimmers. For instance, only a 10th of a second separated the gold and silver medalists in the women’s 50 m freestyle final at the recent 2019 European short-course championships [1]. In search for marginal gains, sophisticated laboratory analyses have investigated kinematic and kinetic mechanisms contributing to swim races [2,3], whereas real race scenarios are used to derive benchmarks and normative data [4,5,6]. In particular, at international swimming competitions, key performance indicators can be derived from top-elite swimmers in highly standardised conditions. Here, environmental factors hardly affect the swimmer’s performance, as water temperature is regulated to 25–28 °C, in-pool current is not allowed to exceed 1.25 m per minute, and pool length cannot vary by more than +0.010 m [7].

Previous studies derived benchmarks from finalists and semi-finalists at major international championships [4,5]. However, lower-ranked swimmers and those aiming to qualify for such swimming competitions require normative data adapted to slower race times. To provide normative data for swimmers of different performance levels, a recent study established benchmarks based on the 10th, 25th, 50th, 75th, and 90th percentiles of all competitors of the European championships. Data of the 10th percentile corresponded to values of the eight finalists, thus still providing benchmarks for swimmers at the highest level [6].

Early swim research in the 1980s and 1990s was mainly endurance-driven, aiming to optimize stroke parameters and investigate metabolic energy contribution [8,9,10,11]. Later developments and the introduction of a new starting block in 2010, i.e., Omega OSB11 with the inclined rear foot support [12], turned the attention to the acyclic phases, i.e., start performance. As such, scientific studies have investigated different starting techniques and on-block force distribution [13,14]. During the start, swimmers push-off a solid base and can fully use the potential of their lower-body strength. Resulting take-off velocities (4.7 ± 0.2 m/s) far beyond free-swimming speed (1.6 ± 0.1 m/s) provide particular potential for the initial race section [2,5].

However, turns provide a more frequent opportunity to push off a solid base, i.e., the pool wall, and initiate each lap with velocities (3.0 ± 0.2 m/s) beyond free-swimming speed [3]. In longer events, with more frequent occurrence of turns and larger time contribution to race time compared to the start (19.8 ± 0.2% vs. 12.3 ± 0.3%, [4]), turns may provide the distinguishing factors in modern swim races. Short-course races (25 m pool length) held during the winter season may further increase the importance of turn performance. More than twice the number of turns for a given event and the repeated push-off from the pool wall increases race time by 2.0 ± 0.6% compared to long-course races [15]. However, as pointed out by a recent review, there is a lack of scientific research regarding short-course swim events [16] in which turn performance may be of particular importance. While most studies evaluated male swimmers or applied a mixed-gender approach, benchmarks and normative data for female swimmers are missing.

From a practical perspective, benchmarks across all swimming strokes and race distances would serve as comparative data when assessing specific key components, i.e., start and turn performances, in routine race and performance analyses of international swimmers [16,17]. With the help of percentiles, performance analysts and coaches may adjust expectations and aims for start and turn performances to the anticipated race time of their swimmers. Quantifying the effect of different race components will help to prioritize skill acquisition, establish training regimes, and determine pacing strategies for competitions [18,19]. Therefore, the aims of the study were to (1) investigate the effect of the acyclic phases, start and turn performance, and its contribution to short-course race time in top-elite female swimmers and (2) provide benchmarks and normative data across all of performance levels of the European Short-Course Championships, for all swimming strokes (butterfly (BU), backstroke (BA), breaststroke (BR), freestyle (FR)), and all race distances (50, 100, 200, 400, 800 m).

## 2. Materials and Methods

### 2.1. Participants

All individual races (*n* = 798) of female competitors (age: 20.6 ± 3.9 yrs, FINA points: 792 ± 78 a.u.) at the 2019 European Short-Course Swimming Championships were monitored by video. Swimmers competing at international events hosted by the European Swimming Association (Ligue Européenne de Natation—LEN) agree to be monitored by video during competitions for television broadcasting and race analysis by the participating nations. The study was pre-approved by the leading institutions internal review board (registration number: 098-LSP-191119) and was in line with the ethical carta of the World Medical Association for research involving human subjects (Helsinki Declaration).

### 2.2. Procedures

**Figure 1:**
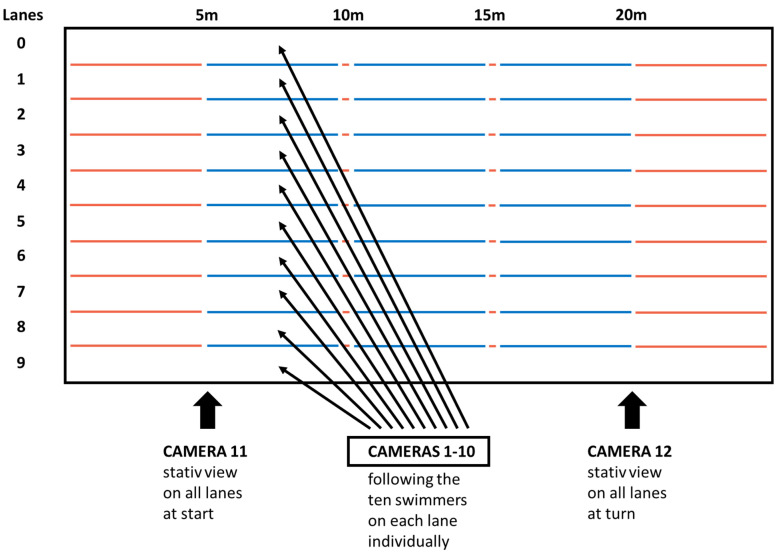
Overview of camera positions during the data collection.

Video footage was collected with a 12 camera system at 50 Hz (Spiideo, Malmö, Sweden). Ten of the cameras followed the ten swimmers on each lane individually, while two static cameras were positioned at 90-degree angle to the lanes and monitored the starts and turns of all swimmers (V59 PTZ, Axis Communications AB, Lund, Sweden; Figure 1). Race times were electronically measured down to a hundredth of a second and provided by the official timekeeper of the championships (Microplus Data Processing and Timing, Marene, Italy).

### 2.3. Data Collection

Using a video analyses software (Kinovea 0.9.1; Joan Charmant & Contrib., https://kinovea.org/, accessed on 10 August 2020), video footage was synchronised to the optical starting signal. The top of the swimmer’s head passing the 5, 10, and 15 m marks of the lane ropes determined start performance. Time from top of the head passing the 5 m mark before wall contact until the 10 m mark after wall contact determined turn performance, i.e., total turn time. For each turn, corresponding split times were determined as the last 5 m prior to wall contact (5 m in), initial 5 m, and 10 m after wall contact (5 m out and 10 m out, respectively). First contact of hands (BU and BR) or feet (BA and FR) at the pool wall determined the end of the lap. Turn performance was assessed based on previously reported breakout distances of 6.6 ± 0.8 m to 10.6 ± 2.1 m for female 100 m [4] as well as 5.5 ± 1.0 m to 8.6 ± 0.5 m for female 200 m BU, BA, BR, and FR races [5], although a 15 m underwater distance would be permitted by official swimming rules [7]. For each individual, mean values for 5 m in, 5 m out, 10 m out, and total turn time across all turns of a race were used for statistical analysis and to compare turn times between race distances as described previously [6].

Time events were manually marked in the analysis software and subsequently exported to an Excel worksheet (Excel 2016, Microsoft Corporation, Redmond, WA, USA) to calculate split times. To determine inter-rater reliability, 5% of the races were randomly selected and analysed in duplicate by a second expert swimming analyst. The 5, 10, and 15 m start times showed an intra-class correlation coefficient (ICC) with corresponding upper and lower bounds of 95% confidence interval of 0.977 (0.956–0.988), 0.999 (0.998–1.000), and 0.988 (0.977–0.993), respectively. Total turn times with corresponding 5 m in, 5 m out, and 10 m out split times revealed an ICC of 1.000 (1.000–1.000), 0.995 (0.990–0.997), 0.992 (0.986–0.996), and 0.999 (0.998–0.999), respectively.

### 2.4. Statistical Analysis

Statistical analyses were performed using JASP version 0.14 (JASP-Team, University of Amsterdam, Amsterdam, The Netherlands). The 10th, 25th, 50th, 75th, and 90th percentiles were calculated across all participants to present normative data for different performance levels. Using a similar approach, the 10th percentile corresponded to mean values of the finalists and provided benchmarks for top-elite swimmers [6]. To investigate the effect of start and turn performance on race time, multiple linear regression analysis was performed with race time as the dependent variable and 15 m start time and mean total turn time as predictors. Additionally, race times were correlated to start and turn times using Pearson’s product moment correlation coefficient with a corresponding 95% confidence interval (CI). Coefficients <0.3, 0.3–0.5, 0.5–0.7, 0.7–0.9, and >0.9 were considered small, moderate, large, very large, and nearly perfect, respectively [20]. An alpha-level <0.05 indicated statistical significance. Values larger than three times the standard deviation were identified as outliers [21]. Missing values were replaced with the nearby mean of that particular heat [21]. In total, 14 missing values and 99 outliers were identified, corresponding to 0.47% of 24,047 raw data points. Due to missing video footage, heats 1–4 of the 50 m BA event were excluded from the 5 m out turn time analysis, and heat 5 of the 200 m BR event was excluded from the start analysis. Based on the standard procedure for large samples sizes, normality was confirmed with residuals and predicted values showing a random pattern around zero in the scatter plot, standardised residuals a Gaussian distribution in the Histogram, and standardised residuals a straight line in the Q-Q plot [21].

## 3. Results

Table 1 and Table 2 provide benchmarks and normative data for start and turn times using the 10th, 25th, 50th, 75th, and 90th percentiles across all female competitors of the European Short-Course Championships. Contribution of start performance to race time was highest for sprint events, i.e., 23.92–27.43%. Contribution of turns to race time increased the longer the race distance and was highest for 800 m FR, i.e., 55.67–56.37%. From 10th to 90th percentile (fastest to slowest swimmers), race times became progressively slower, but the contribution of acyclic phases, i.e., start and turn times, increased. This was the case for all swimming strokes and race distances.

**Figure 2:**
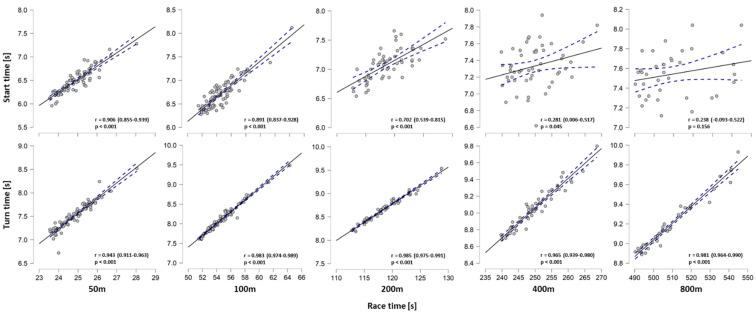
Pearson’s product moment correlation coefficient with 95% confidence interval for 15 m start and turn times (from 5 m before to 10 m after wall contact) with 50, 100, 200, 400, and 800 m freestyle race times across all female competitors of each event.

The mechanistic analysis showed close correlations between total turn time and 50 m to 800 m race time (Figure 2). Start time correlated with 50 m (r = 0.906 (95% CI: 0.855–0.939) *p* < 0.001) but not with 800 m race times (r = 0.238 (95% CI: −0.093–0.522) *p* = 0.156), as correlation coefficients progressively decreased the longer the race distance. The regression model predicted 66 to 98% of total variance in race time based on start and turn performances (Table 3). The effect of start performance on race time decreased the longer the distance. Turn performance showed a larger effect on race time than start performance for 100 m (β = 0.813 and 0.201, respectively) and longer events. However, even for the 50 m BU (β = 0.635 vs. 0.360) and 50 m FR (β = 0.616 vs. 0.391) events, turn performance had a larger effect on race time compared to start performance. Effects were similar for 50 m BR (β = 0.440 vs. 0.433).

## 4. Discussion

The present study provides benchmarks and normative data for start and turn performance with corresponding split times for all performance levels (10th, 25th, 50th, 75th, and 90th percentiles) across the female competitors of the European Short-Course Championships. Normative data were established for all swimming strokes (BU, BA, BR, FR) and race distances (50, 100, 200, 400, 800 m). Mechanistic analysis revealed a greater contribution of start and turn performance to race time in slower swimmers; hence, they lost time during the acyclic phases. Multiple linear regression analysis explained up to 98% of the total variance in race time and showed a larger effect of a turn compared to start performance for all race distances ≥100 m. Even for the 50 m events, turn performance had an equal (BR) or larger effect (BU and FR) on race time compared to start performance.

As high-performance sports strive towards the best possible performance, previous studies derived benchmarks from (semi-)finalists of major international competitions only [4,5]. Additionally, most studies presented normative data for a specific swimming stroke [3,17] or a single race distance [4,5,18]. To provide a complete data base of normative data and make them comparable between swimming strokes and race distances, the present study derived benchmarks from a single population, i.e., female European Short-Course Championship competitors, and analysed all swimming strokes (BU, BA, BR, FR) and race distances (50, 100, 200, 400, 800 m). Additionally, races from all competitors of each event were analysed to establish percentiles from fastest to slowest swimmers. Hereby, the 10th percentile corresponds to data of the finalists, as shown previously [6]. It is important to note that section times and skills, i.e., start and turn performance, vary with corresponding slower race times. Therefore, the 25th, 50th, 75th, and 90th percentile data should be used for swimmers of different performance levels and those aiming to qualify for the European Championships. Coaches may use the present data base to set realistic goals for race sections and skills based on the targeted race time.

In the regression model, which explained up to 98% of total variance in race time, the effect of turn performance increased the longer the race distance. Concurrently, standardised beta coefficients for start performance decreased and became trivial for race distances ≥200 m. Interestingly, for the 50 m BU and FR events involving one turn and one start, the turn performance showed a larger effect on race time than the start performance. This is unexpected as both skills were investigated over the same distance, i.e., 15 m, and initial velocity after the start (4.6 ± 0.3 m/s) is faster than after the turn (3.0 ± 0.2 m/s; [3]). However, the larger time contribution of the turn (~30%) compared to the start (~25%) may provide more potential for fast swimmers to apply technical skills, such as efficient streamlining and underwater butterfly kicking, which, in addition to velocity at take-off and on-wall force production, contribute to turn performance [22].

The time contribution of the acyclic phases, i.e., start and turn, increased from 10th to 90th percentile (fastest to slowest swimmers) across all swimming strokes and race distances. Hence, relative to their faster race time, faster swimmers spend even less time on the acyclic phases and showed superior start and turn performances. Anecdotal evidence discussed by performance analysts suggests that swim velocities have plateaued in recent years; however, top-elite swimmers have continued to improve their start and turn performances. This is supported by Gonjo and co-worker Gonjo and Olstad [16] in a longitudinal analysis showing that breakout distances after starts and turns improved up to 81% during the last three decades depending on swimming stroke and race distance. In contrast, swim velocities improved by less than 5% across two decades [23]. With the increasing focus of scientific research on the acyclic phases [2,3,22], the development of on-land strength and conditioning programs aiming to improve take-off velocities after starts and turns [24], and the large effect in the regression model, turn performance presents an important key performance indicator and may be the distinguishing factor in modern swim races.

During traditional training programs, with a high volume of low-intensity swimming, numerous turns are performed in each session [19,25]. However, timing when approaching the pool wall, body rotation, take-off angle, transition from wall push-off to underwater, and subsequent free-swimming phase are specific to the actual swim velocity [22,26]. In order to develop these kinematic aspects and prepare for competitions, training programs should involve a substantial amount of race-pace-specific drills designed to improve turn performance. Additionally, swimmers can benefit from on-land strength and conditioning programs to develop the specific lower-body power needed for on-wall force production [24,27].

The present investigation assessed turn performance from 5 m before to 10 m after wall contact based on previously reported breakout distances of 5.5 ± 1.0 m to 10.6 ± 2.1 m for the female 100 m and 200 m BU, BA, BR, and FR races [4,5]. However, official swimming rules allow swimmers to remain underwater up to 15 m after each turn [7]. A limitation of the study is that turn times were not determined based on the individualized measurement of breakout distances [28]. In addition to the start and turn performances, benchmarks and normative data are required for stroke and velocity kinetics. Adding the free-swimming phase to the regression model would further help to quantify contributing factors to race time in elite female swimmers. Deriving benchmarks from races at World championships and Olympic games would include other strong swimming nations such as USA and Australia [29] and increase performance level to the highest level.

## 5. Conclusions

The time contribution of the acyclic phases, i.e., start and turn, increased from the 10th to 90th percentile (fastest to slowest swimmers) across all swimming strokes and race distances. Hence, relative to their faster race time, faster swimmer were even faster during the acyclic phases. Additionally, the regression model indicated turn performance as a key performance indicator for modern short-course races, in particular for race distances ≥100 m. To set realistic goals, establish race strategies, and develop training programs, coaches and swimmers should use normative data based on the 10th to 90th percentile depending on their targeted race time. During traditional volume-based training programs, swimmers perform numerous turns at slow velocities. From a practical perspective, and based on the importance of turn performance for race time, swimmers may benefit from race-pace-specific drills aiming to improve the acyclic phases, i.e., turns, as well as on-land strength and conditioning programs to develop the specific lower-body power needed for on-wall force production.

## Figures and Tables

**Table 1:** Percentiles for 15 m *start performance* and its contribution to race time with corresponding 5 m and 10 m split times across all female competitors of the European Short-Course Championships.

	Percentile	Race Time [mm:ss.00]	Start Performance [s]			Contribution [%]
			5 m	10 m	15 m	
**Butterfly**						
**50 m**	**10th**	**00:25.24**	**1.46**	**3.50**	**6.14**	**24.20**
	25th	00:25.55	1.52	3.60	6.32	24.53
	50th	00:26.03	1.54	3.73	6.48	24.99
	75th	00:26.60	1.60	3.91	6.71	25.30
	90th	00:27.27	1.62	4.08	6.96	25.72
**100 m**	**10th**	**00:56.69**	**1.49**	**3.61**	**6.36**	**11.07**
	25th	00:57.09	1.52	3.70	6.44	11.18
	50th	00:58.13	1.56	3.82	6.66	11.45
	75th	00:59.28	1.60	3.95	6.83	11.62
	90th	01:00.55	1.64	4.14	7.23	11.99
**200 m**	**10th**	**02:05.00**	**1.58**	**3.94**	**6.92**	**5.41**
	25th	02:06.48	1.60	3.97	7.00	5.49
	50th	02:07.54	1.64	4.10	7.16	5.58
	75th	02:10.28	1.73	4.26	7.40	5.66
	90th	02:13.35	1.80	4.37	7.49	5.86
**Backstroke**						
**50 m**	**10th**	**00:26.29**	**1.70**	**4.14**	**6.78**	**25.67**
	25th	00:26.58	1.74	4.20	6.93	26.03
	50th	00:26.96	1.80	4.34	7.14	26.37
	75th	00:27.85	1.89	4.55	7.45	26.83
	90th	00:28.95	2.01	4.96	8.01	27.43
**100 m**	**10th**	**00:56.69**	**1.72**	**4.18**	**6.92**	**12.08**
	25th	00:57.48	1.80	4.28	7.12	12.26
	50th	00:58.44	1.87	4.43	7.36	12.50
	75th	00:59.60	1.95	4.61	7.57	12.87
	90th	01:01.50	2.00	4.84	7.94	13.12
**200 m**	**10th**	**02:03.04**	**1.72**	**4.24**	**7.30**	**5.78**
	25th	02:05.09	1.84	4.50	7.49	5.96
	50th	02:07.14	1.94	4.66	7.76	6.05
	75th	02:09.92	2.01	4.79	8.01	6.26
	90th	02:11.08	2.10	4.94	8.20	6.33
**Breaststroke**						
**50 m**	**10th**	**00:29.77**	**1.50**	**3.66**	**7.30**	**23.92**
	25th	00:30.13	1.52	3.76	7.40	24.43
	50th	00:30.57	1.56	3.88	7.60	24.86
	75th	00:31.26	1.62	4.03	7.81	25.22
	90th	00:32.18	1.66	4.27	7.97	25.60
**100 m**	**10th**	**01:05.15**	**1.50**	**3.76**	**7.44**	**11.42**
	25th	01:05.42	1.54	3.88	7.62	11.53
	50th	01:06.24	1.60	3.96	7.78	11.71
	75th	01:08.02	1.62	4.10	8.09	11.90
	90th	01:09.37	1.66	4.24	8.30	12.06
**200 m**	**10th**	**02:20.42**	**1.54**	**3.91**	**7.76**	**5.43**
	25th	02:21.99	1.58	3.96	7.90	5.51
	50th	02:24.45	1.62	4.16	8.08	5.65
	75th	02:26.12	1.68	4.34	8.38	5.76
	90th	02:28.45	1.72	4.50	8.55	5.82
**Freestyle**						
**50 m**	**10th**	**00:23.83**	**1.46**	**3.61**	**6.20**	**25.41**
	25th	00:24.06	1.50	3.66	6.26	25.84
	50th	00:24.76	1.52	3.78	6.46	26.11
	75th	00:25.38	1.59	3.98	6.64	26.36
	90th	00:25.90	1.63	4.17	6.88	26.57
**100 m**	**10th**	**00:52.23**	**1.48**	**3.66**	**6.38**	**12.00**
	25th	00:53.19	1.52	3.76	6.46	12.11
	50th	00:54.27	1.58	3.94	6.68	12.30
	75th	00:55.47	1.64	4.08	6.86	12.52
	90th	00:57.01	1.69	4.26	7.09	12.62
**200 m**	**10th**	**01:54.95**	**1.57**	**3.90**	**6.73**	**5.77**
	25th	01:55.86	1.62	4.06	6.86	5.85
	50th	01:58.08	1.64	4.18	7.10	5.96
	75th	02:00.62	1.68	4.32	7.22	6.06
	90th	02:03.04	1.74	4.41	7.36	6.18
**400 m**	**10th**	**04:01.85**	**1.62**	**4.04**	**7.00**	**2.80**
	25th	04:04.07	1.66	4.19	7.19	2.87
	50th	04:08.64	1.68	4.32	7.30	2.96
	75th	04:12.79	1.76	4.47	7.51	3.01
	90th	04:18.40	1.80	4.60	7.62	3.08
**800 m**	**10th**	**08:13.40**	**1.66**	**4.18**	**7.28**	**1.39**
	25th	08:16.07	1.70	4.32	7.32	1.45
	50th	08:24.97	1.80	4.50	7.56	1.49
	75th	08:39.31	1.82	4.64	7.68	1.52
	90th	08:57.47	1.87	4.70	7.79	1.54

**Table 2:** Percentiles for *turn time* (from 5 m before to 10 m after wall contact) and its contribution to race time with corresponding 5 m in, 5 m out, and 10 m out split times across all female competitors of the European Short-Course Championships.

	Percentile	Turn Performance [s]				Contribution [%]
		5 m In	5 m Out	10 m Out	Total Turn Time	
**Butterfly**						
**50 m**	**10th**	**2.48**	**2.62**	**5.34**	**7.96**	**30.94**
	25th	2.55	2.70	5.44	8.04	31.09
	50th	2.61	2.81	5.52	8.11	31.31
	75th	2.72	2.86	5.70	8.33	31.53
	90th	2.80	2.96	5.82	8.50	31.73
**100 m**	**10th**	**2.80**	**2.77**	**5.67**	**8.53**	**44.88**
	25th	2.83	2.82	5.80	8.66	45.07
	50th	2.87	2.90	5.89	8.74	45.26
	75th	2.94	2.95	6.02	8.93	45.53
	90th	3.01	3.06	6.21	9.17	45.87
**200 m**	**10th**	**3.05**	**2.98**	**6.23**	**9.39**	**51.59**
	25th	3.10	3.05	6.33	9.48	52.05
	50th	3.16	3.12	6.40	9.54	52.34
	75th	3.22	3.21	6.60	9.73	52.60
	90th	3.31	3.29	6.72	9.99	52.74
**Backstroke**						
**50 m**	**10th**	**3.10**	**1.73**	**4.51**	**7.64**	**28.80**
	25th	3.12	1.78	4.60	7.73	28.97
	50th	3.18	1.86	4.72	7.88	29.19
	75th	3.29	1.93	4.95	8.17	29.48
	90th	3.37	2.01	5.16	8.51	29.70
**100 m**	**10th**	**3.28**	**1.93**	**4.84**	**8.15**	**42.60**
	25th	3.31	1.95	4.94	8.26	43.01
	50th	3.38	2.01	5.04	8.43	43.26
	75th	3.48	2.08	5.24	8.70	43.52
	90th	3.56	2.17	5.36	8.88	43.85
**200 m**	**10th**	**3.54**	**1.99**	**5.18**	**8.74**	**49.49**
	25th	3.58	2.05	5.34	8.92	49.74
	50th	3.63	2.11	5.46	9.12	50.23
	75th	3.71	2.20	5.59	9.27	50.51
	90th	3.75	2.27	5.73	9.43	50.65
**Breaststroke**						
**50 m**	**10th**	**2.99**	**2.67**	**6.06**	**9.13**	**29.90**
	25th	3.04	2.74	6.20	9.24	30.39
	50th	3.10	2.78	6.32	9.39	30.64
	75th	3.18	2.85	6.40	9.54	30.97
	90th	3.24	2.91	6.51	9.66	31.23
**100 m**	**10th**	**3.19**	**2.79**	**6.36**	**9.65**	**43.92**
	25th	3.22	2.85	6.49	9.75	44.30
	50th	3.29	2.93	6.59	9.88	44.66
	75th	3.38	3.01	6.75	10.14	45.05
	90th	3.49	3.11	6.92	10.32	45.37
**200 m**	**10th**	**3.49**	**2.96**	**6.71**	**10.27**	**50.72**
	25th	3.55	3.01	6.79	10.36	50.94
	50th	3.62	3.08	6.95	10.59	51.26
	75th	3.68	3.18	7.09	10.70	51.57
	90th	3.74	3.24	7.24	10.96	51.84
**Freestyle**						
**50 m**	**10th**	**2.78**	**1.73**	**4.33**	**7.18**	**29.87**
	25th	2.82	1.80	4.44	7.28	30.10
	50th	2.88	1.86	4.62	7.46	30.26
	75th	2.96	1.96	4.76	7.70	30.47
	90th	3.06	2.03	4.86	7.82	30.66
**100 m**	**10th**	**2.98**	**1.88**	**4.73**	**7.75**	**44.12**
	25th	3.02	1.97	4.85	7.87	44.32
	50th	3.08	2.06	4.97	8.05	44.44
	75th	3.17	2.11	5.07	8.24	44.63
	90th	3.24	2.18	5.21	8.46	44.82
**200 m**	**10th**	**3.22**	**2.03**	**5.08**	**8.37**	**50.83**
	25th	3.28	2.09	5.18	8.46	50.99
	50th	3.36	2.15	5.31	8.65	51.14
	75th	3.45	2.21	5.39	8.86	51.33
	90th	3.52	2.27	5.51	9.00	51.55
**400 m**	**10th**	**3.37**	**2.09**	**5.32**	**8.75**	**53.88**
	25th	3.43	2.16	5.41	8.83	54.12
	50th	3.51	2.23	5.50	9.03	54.36
	75th	3.62	2.27	5.61	9.17	54.63
	90th	3.68	2.36	5.72	9.37	54.83
**800 m**	**10th**	**3.45**	**2.12**	**5.42**	**8.92**	**55.67**
	25th	3.49	2.19	5.50	8.98	55.79
	50th	3.57	2.24	5.55	9.13	55.96
	75th	3.66	2.29	5.75	9.38	56.19
	90th	3.80	2.41	5.80	9.65	56.37

**Table 3:** Effect of 15 m start and turn times (from 5 m before to 10 m after wall contact) on race time across all female competitors of the European Short-Course Championships using multiple linear regression analysis.

	Regression Model				Regression Coefficients			
	Entries	R Square	F Value	*p*-Value		Standar-Dised Beta	T Value	*p*-Value
**Butterfly**								
**50 m**	60	**0.95**	*F*_(2|57)_ = 522	*p* < 0.001	Start Turn	**0.360** **0.635**	*T* = 4.95 *T* = 8.74	*p* < 0.001 *p* < 0.001
**100 m**	56	**0.87**	*F*_(2|53)_ = 173	*p* < 0.001	Start Turn	**−0.041** **0.962**	*T* = −0.53 *T* = 12.42	*p* = 0.597 *p* < 0.001
**200 m**	30	**0.91**	*F*_(2|27)_ = 128	*p* < 0.001	Start Turn	**−0.064** **0.988**	*T* = −0.86 *T* = 13.30	*p* = 0.397 *p* < 0.001
**Backstroke**								
**50 m**	59	**0.97**	*F*_(2|56)_ = 901	*p* < 0.001	Start Turn	**0.550** **0.446**	*T* = 6.83 *T* = 5.53	*p* < 0.001 *p* < 0.001
**100 m**	60	**0.94**	*F*_(2|57)_ = 416	*p* < 0.001	Start Turn	**0.167** **0.819**	*T* = 2.45 *T* = 12.05	*p* = 0.017 *p* < 0.001
**200 m**	31	**0.91**	*F*_(2|28)_ = 144	*p* < 0.001	Start Turn	**−0.244** **1.111**	*T* = −3.06 *T* = 13.95	*p* = 0.005 *p* < 0.001
**Breaststroke**								
**50 m**	68	**0.66**	*F*_(2|65)_ = 64	*p* < 0.001	Start Turn	**0.433** **0.440**	*T* = 4.01 *T* = 4.08	*p* < 0.001 *p* < 0.001
**100 m**	79	**0.83**	*F*_(2|76)_ = 187	*p* < 0.001	Start Turn	**0.171** **0.775**	*T* = 2.35 *T* = 10.66	*p* = 0.021 *p* < 0.001
**200 m**	39	**0.86**	*F*_(2|36)_ = 110	*p* < 0.001	Start Turn	**0.043** **0.904**	*T* = 0.59 *T* = 12.42	*p* = 0.556 *p* < 0.001
**Freestyle**								
**50 m**	77	**0.94**	*F*_(2|74)_ = 536	*p* < 0.001	Start Turn	**0.391** **0.616**	*T* = 7.29 *T* = 11.49	*p* < 0.001 *p* < 0.001
**100 m**	85	**0.98**	*F*_(2|82)_ = 1823	*p* < 0.001	Start Turn	**0.201** **0.813**	*T* = 6.47 *T* = 26.21	*p* < 0.001 *p* < 0.001
**200 m**	56	**0.97**	*F*_(2|53)_ = 885	*p* < 0.001	Start Turn	**−0.004** **0.988**	*T* = -0.13 *T* = 29.51	*p* = 0.901 *p* < 0.001
**400 m**	51	**0.94**	*F*_(2|48)_ = 374	*p* < 0.001	Start Turn	**−0.104** **1.004**	*T* = -2.70 *T* = 26.17	*p* = 0.010 *p* < 0.001
**800 m**	37	**0.97**	*F*_(2|34)_ = 554	*p* < 0.001	Start Turn	**−0.090** **1.011**	*T* = -2.87 *T* = 32.30	*p* = 0.007 *p* < 0.001

## Data Availability

All data are available on request by the corresponding author.
